# Destabilizing an interacting motif strengthens the association of a designed ankyrin repeat protein with tubulin

**DOI:** 10.1038/srep28922

**Published:** 2016-07-06

**Authors:** Shoeb Ahmad, Ludovic Pecqueur, Birgit Dreier, Djemel Hamdane, Magali Aumont-Nicaise, Andreas Plückthun, Marcel Knossow, Benoît Gigant

**Affiliations:** 1 Institute for Integrative Biology of the Cell (I2BC), CEA, CNRS, Univ Paris-Sud, Université Paris-Saclay, 91198, Gif-sur-Yvette cedex, France; 2 Department of Biochemistry, University of Zurich, Winterthurerstrasse 190, CH-8057, Zurich, Switzerland; 3 Laboratoire de Chimie des Processus Biologiques, CNRS-UMR 8229, Collège De France, 11 place Marcelin Berthelot, 75231 Paris Cedex 05, France

## Abstract

Affinity maturation by random mutagenesis and selection is an established technique to make binding molecules more suitable for applications in biomedical research, diagnostics and therapy. Here we identified an unexpected novel mechanism of affinity increase upon *in vitro* evolution of a tubulin-specific designed ankyrin repeat protein (DARPin). Structural analysis indicated that in the progenitor DARPin the C-terminal capping repeat (C-cap) undergoes a 25° rotation to avoid a clash with tubulin upon binding. Additionally, the C-cap appears to be involved in electrostatic repulsion with tubulin. Biochemical and structural characterizations demonstrated that the evolved mutants achieved a gain in affinity through destabilization of the C-cap, which relieves the need of a DARPin conformational change upon tubulin binding and removes unfavorable interactions in the complex. Therefore, this specific case of an order-to-disorder transition led to a 100-fold tighter complex with a subnanomolar equilibrium dissociation constant, remarkably associated with a 30% decrease of the binding surface.

The selection of binders for applications where high affinity and specificity are needed has long been based on the use of the immune system. In the last ca. 15 years, as an alternative to natural and synthetic antibodies, libraries of artificial proteins have been developed as a resource of potential binders^1^,^2^. These libraries are typically based on a conserved, well-defined, protein scaffold and on a variable binding surface. Such binders often extend the possibilities of antibodies, e.g. with cysteine-free scaffolds that are able to work under reducing conditions. Using molecular display technologies (e.g. ribosome display), binders for a particular target are selected from the library. Once first generation binders have been identified, steps of diversification and selection may be applied to obtain higher affinity ones^3^, a process reminiscent of the maturation of antibodies during the immune response.

The binding of a ligand, where the ligand may be a macromolecule (protein, nucleic acid…) or a small molecule, can affect a protein in many ways^4^. In addition to small local changes, domain movements may occur in an induced fit mechanism. More dramatic rearrangements like disorder-to-order transitions are also frequently observed^5^. The reverse case, an order-to-disorder transition, has also been documented^6^. In this case, the binding surface is typically hidden in the folded protein and becomes more accessible after melting of a secondary structural element or of a domain^7^. As to the unfolding of a protein motif that is part of a protein−ligand interface, it is expected to lead to the destabilization of the assembly.

In contrast to this last expectation, we report a case where a gain in affinity results from the destabilization of a two-helix-containing motif. We previously selected artificial binders to the αβ tubulin heterodimer (tubulin) from a library of designed ankyrin repeat proteins (DARPins)^8^,^9^. Here we applied cycles of randomization alternately with selection steps in order to identify higher affinity binders. This strategy led to evolved DARPins that bind tubulin two orders of magnitude stronger than the parental one, resulting in complexes with subnanomolar dissociation constant. Biochemical and structural characterizations demonstrated that the affinity increase is coupled with the destabilization of the ankyrin C-terminal capping motif (C-cap) of the DARPin, which in the parent DARPin interacts with tubulin but needs to undergo a rotation to avoid a clash. This remarkable order-to-disorder transition illustrates a new mechanism for affinity maturation.

## Results

### DARPins with an improved affinity for tubulin

In the course of the study of microtubules, we have selected DARPins that bind tubulin as multipurpose tools and in particular as crystallization chaperones^9^. Whereas these DARPins have proven instrumental to crystallize tubulin and its complexes with interacting proteins (e.g. see ref. 10), the tubulin−DARPin affinity is within the range of values usually observed for interactions that are transient on a time scale of seconds to minutes^4^, the measured equilibrium dissociation constant (K_D_) being in the 100 nM range^9^. Beyond their use as crystallization chaperones, which usually occur at high concentrations, other applications, for example in studies within living cells^11^,^12^, would benefit from higher affinity binders.

**Figure 1:**
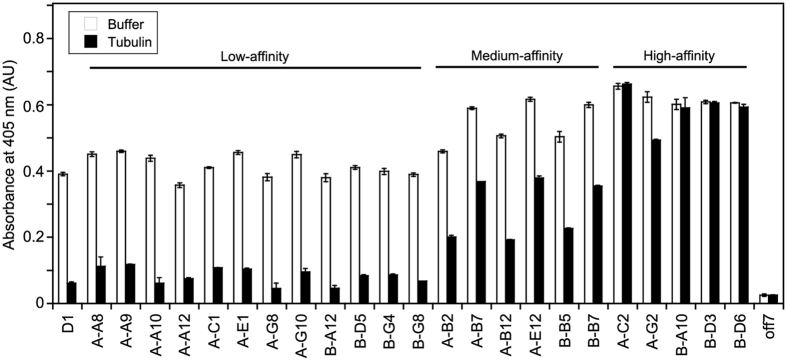
ELISA analysis of D1 variants after two rounds of error-prone PCR followed by selection using ribosome display. DARPins at a 30 nM concentration were incubated in 10 nM biotinylated peptide-coupled tubulin-coated wells. For off-rate estimation, after DARPin binding and the washing steps, the wells were incubated either with buffer or with a 100 nM tubulin solution, to prevent the rebinding of detached DARPins to immobilized tubulin. The DARPins were ranked as low-, medium- or high-affinity binders. D1 is the parent DARPin whereas off7 is an unrelated one^8^, not binding to tubulin and used as a negative control. Error bars are standard deviations from duplicate experiments. AU, absorbance unit.

To this end we evolved the tubulin-binding DARPin named D1 by applying cycles of error-prone PCR-based diversification followed by selection using ribosome display. The selected variants were further screened in an ELISA-based assay. In this assay, tubulin covalently linked to a biotinylated stathmin-like peptide^9^,^13^ was immobilized on a neutravidin-coated plate, which was then incubated with DARPin variants. Finally, the wells were incubated either with buffer alone or with free tubulin in order to trap dissociated DARPins and prevent rebinding to immobilized tubulin. This procedure should lead to the identification of DARPins with a low dissociation rate. As expected^14^ the differentiation was far more pronounced after incubation with free tubulin as competitor of rebinding, allowing us to rank the variants in three categories: those that displayed the lowest signal in the ELISA experiment and behaved essentially like the parent DARPin (the signal being within less than a factor of 2 of that of D1) and those exhibiting a medium signal (3- to 6-fold that of D1) or a high signal (more than about 8-fold that of D1) (Fig. 1).

**Figure 2:**
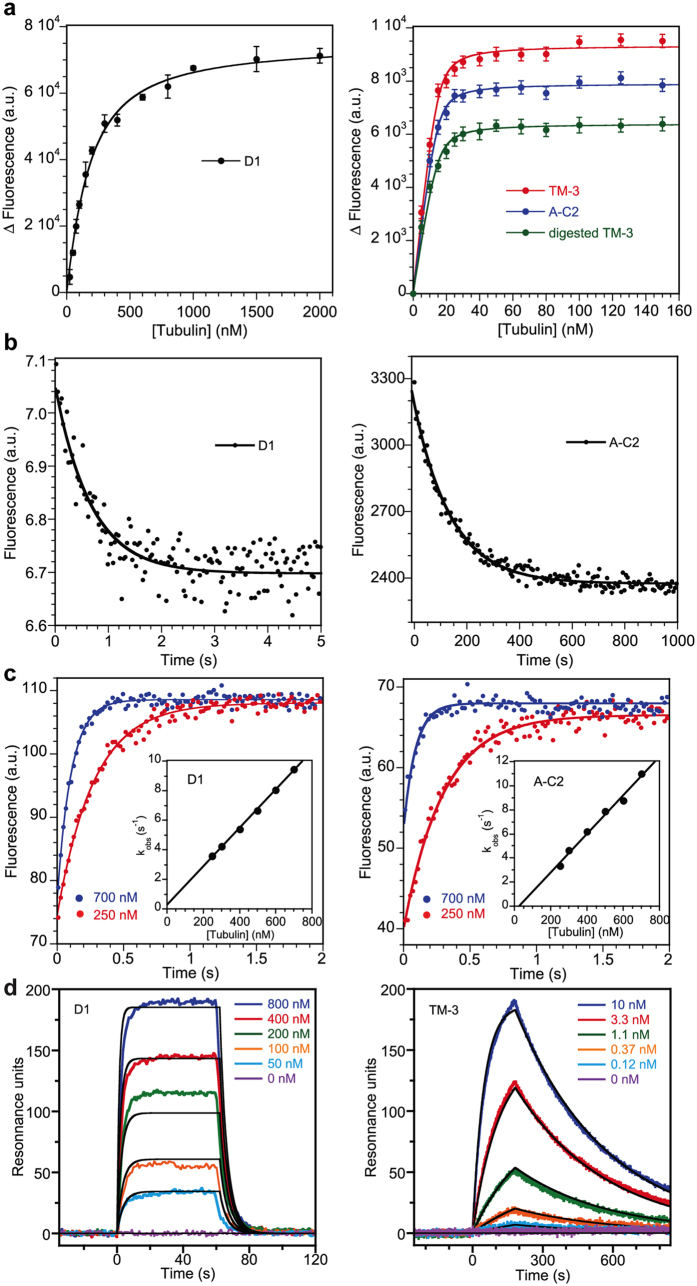
The tubulin-DARPin interaction monitored by fluorescence spectroscopy and surface plasmon resonance (SPR). (**a**) Fluorescence variation of 100 nM (left) or 15 nM (right) acrylodan-labeled DARPins as a function of tubulin concentration. The curve is the fit of the experimental points with Equation 1, from which the K_D_ is extracted. Error bars correspond to standard deviation from duplicate experiments. a.u., arbitrary units. (**b**) Dissociation of acrylodan-labeled DARPin from tubulin. In the case of D1 (left), 5.5 μM unlabeled D1 was added to a 100 nM labeled D1 and 0.5 μM tubulin mixture. The decrease in fluorescence signal was monitored in a stopped-flow apparatus (30% of the data points are shown). In the case of A-C2 (right), 2 μM unlabeled A-C2 was added to a 20 nM labeled A-C2 and 40 nM tubulin mixture and the fluorescence signal was monitored in a spectrofluorometer. The curve is the fit of the experimental points with a mono-exponential decay equation (Equation 2). (c) Determination of the association rate constant by fluorescence. Tubulin at the indicated concentrations was added to a fixed concentration (50 nM) of labeled D1 (left) or A-C2 (right) in a stopped-flow apparatus (20% of the experimental points are displayed). The data were fitted according to Equation 3. The variation of k_obs_ as a function of tubulin concentration is shown in inset, from which the k_on_ (slope of the curve) is derived. (**d**) The tubulin-DARPin interaction monitored by SPR. D1 (left) and TM-3 (right) were immobilized through their His-tag on the sensor chip. Tubulin at the indicated concentration was applied at time zero for 60 s (D1) or 180 s (TM-3), followed by a washing buffer flow. The black curves are the fit of the experimental data using the Langmuir analysis, from which the k_on_ and k_off_ are extracted. In the case of D1, plotting the values at the plateau as a function of the tubulin concentration provided an estimate of the K_D_ (Supplementary Fig. 5) that is very similar to the k_off_/k_on_ ratio.

We wanted to characterize the affinity maturation process further and selected for this purpose A-C2, the DARPin variant which gives the highest signal in the ELISA conditions. First, we quantified by fluorescence spectroscopy the interaction with tubulin of both D1 and A-C2 using acrylodan-labeled cysteine variants. The stepwise addition of tubulin to labeled DARPins led to an increase of the acrylodan fluorescence signal (Fig. 2a). Fitting the data with a quadratic equation (Equation 1, see “Methods”) yielded the value of K_D_ and indicated a change by two orders of magnitude of this value to less than 1 nM in the case of A-C2, down from ~125 nM in the case of D1 (Table 1), the latter value being in excellent agreement with the one determined previously by fluorescence anisotropy^9^. We also determined the association (k_on_) and dissociation (k_off_) rate constants of the tubulin-DARPin complexes. A chase of labeled DARPin from tubulin by the unlabeled counterpart yielded the k_off_ value (Fig. 2b) whereas the k_on_ value was derived from association kinetics under pseudo-first-order conditions (Fig. 2c). In all cases, data were fitted with monoexponential equations (see “Methods”) without any indication of more complex reaction schemes. As expected, the data indicated a dissociation of A-C2 from tubulin two orders of magnitude slower than that of the parent DARPin D1 (Table 1). In contrast, the k_on_ values of the tubulin complexes with D1 and with A-C2 were similar. This scheme is typical for affinity maturation processes^14^,^15^ and results in this case in a subnanomolar K_D_.

### Assessing the contribution of the residues mutated in A-C2

**Figure 3:**
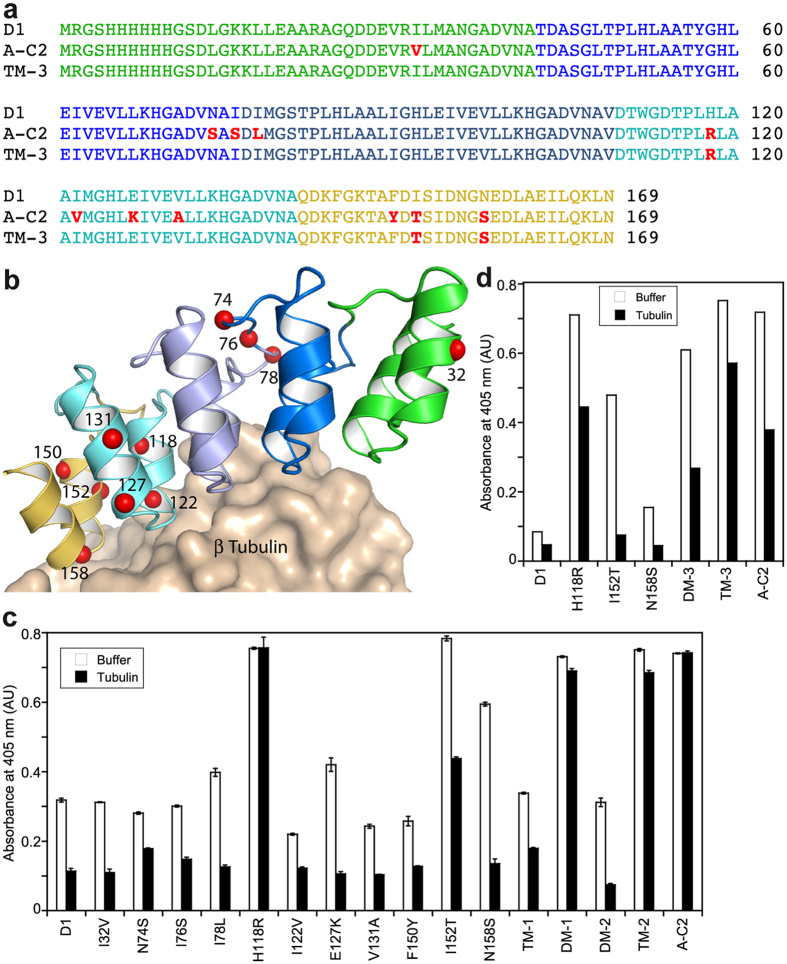
Assessing the contribution of the D1 residues mutated in A-C2. (**a**) Sequence alignment of D1, A-C2 and the optimized TM-3 DARPin (color code: N-cap, green; 1^st^ internal repeat, bright blue, 2^nd^ internal repeat, dark blue; 3^rd^ internal repeat, cyan; C-cap, yellow; mutated residues relative to D1, red). (**b**) Structure of tubulin−D1^9^ with the Cα position of the residues mutated in A-C2 highlighted as red spheres. D1 is colored according to panel A. (**c**) ELISA analysis (binding and off-rate estimation) of the 11 D1 single mutants, 2 double mutants (DM-1: H118R, I122V; DM-2: E127K, V131A) and of 2 triple mutants (TM-1: N74S, I76S, I78L; TM-2: F150Y, I152T, N158S). The experimental conditions were as in Fig. 1. (**d**) ELISA analysis of the DM-3 double mutant (I152T, N158S) and of the TM-3 triple mutant (with the additional H118R substitution) together with the corresponding single mutants. The experimental conditions were as in panel C, except that the concentration of biotinylated peptide-coupled tubulin used for coating was decreased 5-fold (down to 2 nM).

Several mechanisms may come into play to account for the affinity increase of a protein for a ligand. An obvious one is a substitution at the protein−ligand interface that strengthens the interactions with neighboring chemical partners. To determine the contribution of this mechanism to the affinity maturation process of D1, we compared the sequence of A-C2 with that of D1 (Fig. 3a). There were changes at 11 amino acid positions, two of them (I122V and N158S) concerning residues that interact directly with tubulin in the tubulin−D1 complex, taking a 4 Å distance cutoff (Fig. 3b). To investigate the contribution of these two residues as well as those of the other 9 more distant ones, we prepared 11 single mutants of D1, each having one of the 11 substitutions found in A-C2. We also prepared four double or triple mutants comprising mutations that are close in the sequence and are more likely to operate in synergy. We evaluated these constructs for their tubulin-binding ability by ELISA and compared them with D1 and A-C2 (Fig. 3c). The ELISA conditions were the same as those initially used to screen the randomized D1 variants, i.e. the DARPins were first bound to immobilized tubulin then incubated with buffer or with a 100 nM tubulin solution to prevent rebinding, which thus constitutes an off-rate ranking. The strongest effect was obtained with the H118R variant, which behaved as A-C2 in this assay. This effect was confirmed with the H118R, I122V (DM-1) double mutant. The I152T mutation gave an intermediate signal, whereas its combination with F150Y and N158S in the TM-2 construct led to a DARPin as efficient as A-C2 or H118R. It should be noted that the signal of I122V is equivalent to that of the parent DARPin D1 and that a slight effect of the N158S substitution could be detected, but only under the conditions of buffer alone (no tubulin used to prevent rebinding). Therefore, the mutation of these two residues (Ile122 and Asn158), which are at the interface with tubulin, does not contribute much to the higher affinity of A-C2 compared to that of D1.

We then prepared constructs with different combinations of the beneficial mutations and analyzed them in a more stringent ELISA experiment, in which the concentration of modified (biotinylated) tubulin incubated on the neutravidin-coated plate was decreased 5-fold (2 nM instead of 10 nM) (Fig. 3d). With 100 nM non-biotinylated tubulin used to trap dissociated DARPin, the I152T and N158S single mutants were hardly distinguishable from D1, whereas the I152T, N158S double mutant (DM-3) gave a signal higher than that of D1, suggesting a synergistic effect of these two residues. Finally, the introduction of H118R substitution into the TM-3 H118R, I152T, N158S construct (Fig. 3a) led to a DARPin with a signal slightly above that of A-C2 and of H118R (Fig. 3d). To confirm the ELISA results, we quantified by fluorescence spectroscopy the association with tubulin of an acrylodan-labeled TM-3 variant (Fig. 2a, Table 1) and found that TM-3 behaved essentially as A-C2.

### Structural investigation of the affinity maturation process

**Figure 4:**
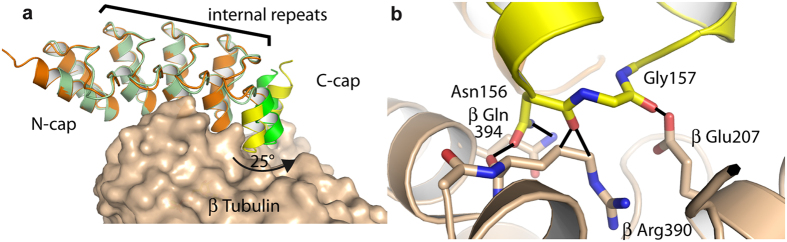
Conformational changes of D1 upon tubulin binding. (**a**) Overview. Uncomplexed D1 (in orange, C-cap in yellow) has been superimposed on D1 in tubulin−D1 (D1 in green with the C-cap in brighter color, β tubulin in beige), taking the N-cap and the internal repeats as a reference. (**b**) Close-up (only β tubulin and uncomplexed D1 are shown). Without a C-cap rotation, its residues would clash with tubulin. Five distances shorter than 2 Å are highlighted as black solid lines.

Since the substitutions at the protein−ligand interface did not seem to have much of an effect on dissociation rate, another mechanism may account for the higher affinity of an evolved binder, constituting the stabilization of the DARPin’s unbound form in a “ready-to-interact” conformation, the one adopted in the complex. This scenario implies that the structure of the isolated parent binder is not optimized for interaction. In this case either an induced fit occurs upon ligand binding or different conformations of the binder coexist and only a sparsely populated one is able to interact^16^,^17^. To investigate this possibility, and despite the fact that DARPins are generally very rigid proteins^18^, we first determined the crystal structure of uncomplexed D1 (Supplementary Table 1) and compared it with that of D1 in complex with tubulin^9^. This comparison indicated that this DARPin indeed changes conformation upon binding (Fig. 4a). With respect to the N-terminal and internal ankyrin repeats, the 2 C-terminal helices rotate by 25° in the complex, thereby preventing a clash with tubulin (Fig. 4b).

**Figure 5:**
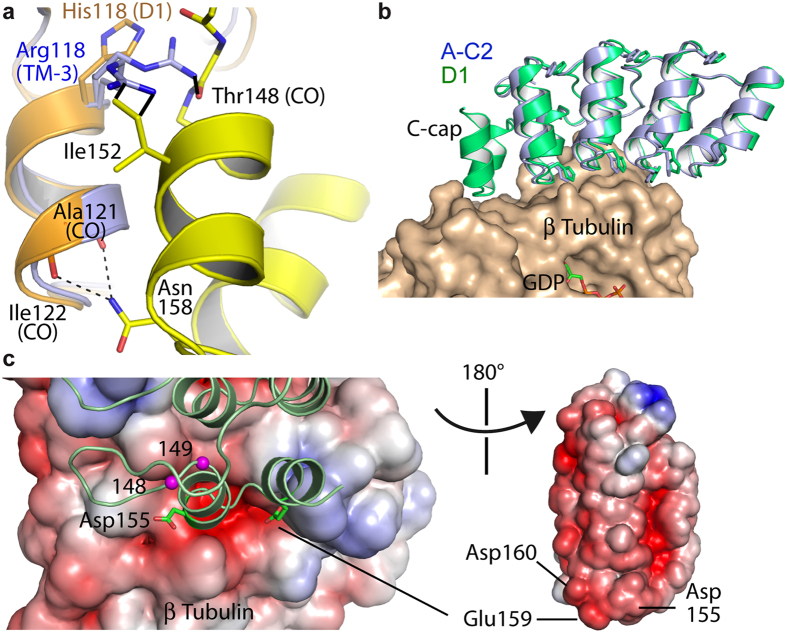
The C-cap motif of the high affinity DARPins is mobile in the crystal structure. (**a**) The H118R mutation destabilizes the C-cap. The TM-3 structure (blue) was superimposed to uncomplexed D1, colored as in Fig. 4a. The Arg118 side chain adopts two alternate conformations in TM-3 (Supplementary Fig. 6). Both would clash with the C-cap if it were folded as in D1 (3 distances shorter than 1.5 Å are highlighted as black solid lines), as would the many other Arg118 conformations we modeled. (**b**) Comparison of the complexes of tubulin (beige) with D1 (green) and with A-C2 (blue). The β tubulin subunits have been superimposed. Some side chains are drawn to help visualize the shift between the DARPins. (**c**) View of the tubulin−D1 interface colored by electrostatic potential. (Left) Electrostatic potential surface of β tubulin (red, negative; blue, positive) with bound D1 (green). The side chains of two acidic residues of the C-cap that are close to an acidic part of the surface of tubulin are shown. The Cαs of residues 148 and 149 are also highlighted as magenta spheres. Subtilisin cleaves the affinity-improved DARPins preferentially after these two residues (see text and Supplementary Fig. 2). (Right) Electrostatic potential surface of D1 centered on the tubulin-interacting surface. Note that the two panels are not to scale.

We next determined the structure of a high-affinity DARPin (namely the TM-3 triple mutant), not bound to tubulin (Supplementary Table 1). These data should allow us to establish whether the evolved DARPins adopt in solution the conformation of tubulin-bound D1. The structure of TM-3 could be determined by molecular replacement taking D1 coordinates as a search probe, but only after removal of the C-cap from the model. There were two molecules of TM-3 in the asymmetric unit. For both, there was no electron density for the C-cap. A comparison with D1 gave an explanation for this observation. The longer side chain of the Arginine residue at position 118 in TM-3 compared to that of a Histidine in D1 led to an interference with the C-cap, hence to the destabilization of this module in TM-3, making it mobile and thus invisible in the X-ray structure (Fig. 5a). However, the comparison with the structure of D1 also indicated that the C-cap would clash with neighboring molecules in the TM-3 crystal if it were folded as in D1, leaving the possibility that different conformations of TM-3 coexist in solution but that only the one with a destabilized (mobile) C-cap is compatible with this crystal packing.

Therefore, we also determined the structure of the evolved DARPin A-C2 in complex with tubulin (Supplementary Table 1). The comparison of this structure with that of tubulin-D1 indicated that the structure of the complex did not change much with respect to the DARPin considered (root mean square deviation (r.m.s.d.) between tubulin−D1 and tubulin−A-C2: 0.62 Å (967 Cαs compared)). Only a slight difference was detected in the DARPin orientation: once the tubulin β subunits of tubulin−A-C2 and of tubulin−D1 have been superimposed, an additional 3 to 4° rotation was needed to superimpose the DARPin moieties of the complexes (Fig. 5b). Actually, the main difference between the two complexes is that in tubulin−A-C2 the two C-terminal helices forming the C-cap motif were not defined in the structure, as in uncomplexed TM-3. However, in contrast to the structure of isolated TM-3, an ordered C-cap would generate only few clashes with symmetry-related molecules in the tubulin−A-C2 crystal; these clashes could be avoided by a different side chain rotamer of two residues.

Taken together, these results indicate that a destabilized C-cap is a characteristic of the evolved DARPins that contributes to the higher affinity for tubulin. In addition to different interaction strengths that might result from the slight DARPin reorientation on tubulin (Fig. 5b), the main structural feature that may explain this affinity difference is an unfavorable contribution of the C-cap in tubulin−D1. Indeed, the tubulin−D1 interface colored by electrostatic potential suggests that there are repulsive charge interactions involving the C-cap: the two C-terminal helices of D1 comprise 5 acidic residues, two of them (Asp155 and Glu159) being close to tubulin and in the vicinity of a mostly acidic cavity (Fig. 5c). The faster dissociation of D1 from tubulin compared to that of the evolved DARPins (Table 1) is well consistent with this scheme. Remarkably, because the C-cap interacts with tubulin in tubulin−D1 (Figs 3b and 4a, and 5b,c)^9^, the total surface buried at the tubulin−DARPin interface calculated from the structures went from ~1825 Å^2^ in the D1 complex down to ~1260 Å^2^ in the case of the A-C2 complex. Therefore, going from tubulin−D1 to tubulin−A-C2, an order-to-disorder transition led to a gain in affinity associated with a ~30% decrease of the buried surface area.

A conformational change upon complex formation, as is the case of D1 upon tubulin binding (Fig. 4a), is expected to limit the association rate constant^19^. In contrast to this expectation, the k_on_ value of acrylodan-labeled D1 was intriguingly high and moreover close to those of the labeled evolved DARPins (Table 1). To ascertain the kinetic parameters determined by fluorescence, we used an orthogonal method and studied the tubulin-DARPin interaction by surface plasmon resonance (SPR). DARPins were immobilized via their N-terminal his-tag on a Ni^2+^-activated tris-NTA sensor chip. The association rate constants were evaluated by injecting different concentrations of tubulin while the subsequent injection of buffer provided an estimate of the dissociation rate constants (Fig. 2d). Whereas the absolute values differed slightly, the SPR results confirmed the trends observed with the fluorescence data. They confirmed in particular that the affinity for tubulin of A-C2 and TM-3 is two orders of magnitude higher than that of D1, and that the main contribution in this affinity increase comes from the k_off_ term (Table 1). Even though the k_on_ values are one order of magnitude lower than those estimated by fluorescence, the SPR data also confirmed that D1 and the affinity-improved DARPins share similar association rate constants for tubulin binding. The reasons for this last observation are not clear at present. One possibility is that the C-cap in D1 is associated more weakly than normal due to long-range repulsive interactions with randomized residues so that the rotation of the C-cap required for binding is very fast.

### The C-terminal capping motif of the high affinity DARPins is disordered in solution

**Figure 6:**
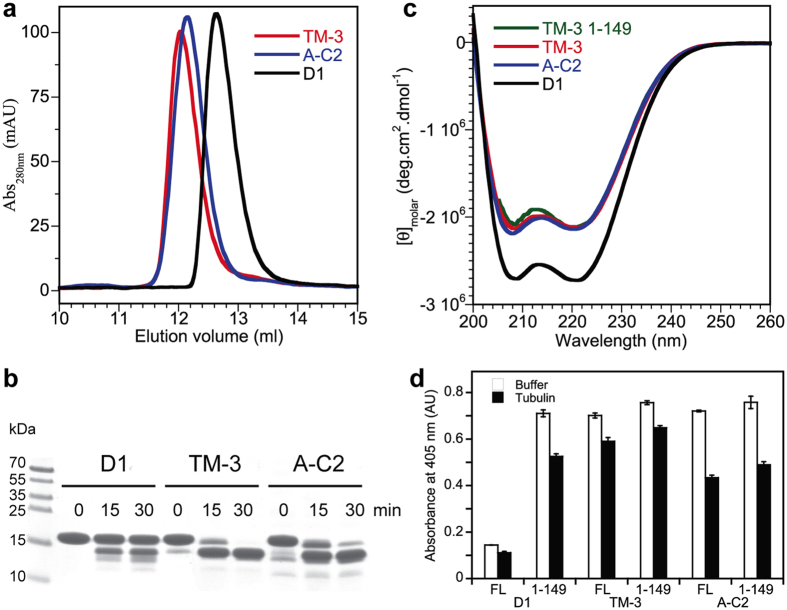
The C-cap motif of the high-affinity DARPins is disordered. (**a**) Gel filtration analysis of 100 μM D1, A-C2 and TM-3. (**b**) SDS-PAGE analysis of the limited proteolysis of D1, TM-3 and A-C2 by subtilisin. DARPins at 40 μM concentration were incubated at 25 °C with subtilisin at a 1:2000 protease:DARPin molar ratio at the indicated times. (**c**) Far-UV circular dichroism spectra of 20 μM D1, A-C2 and TM-3 and of 5 μM TM-3 1-149. The spectra were recorded using a 1 mm path length cuvette. They were normalized and are depicted as molar ellipticity. (**d**) ELISA analysis (tubulin binding and off-rate estimation) of D1, A-C2 and TM-3 along with their C-cap-truncated variants (FL, full length; 1-149, DARPin terminating after residue 149). The experimental conditions were as in Fig. 3d. Here, as in Figs 1 and 3c,d, soluble tubulin at a 100 nM concentration was added after the washing steps to prevent the rebinding of dissociated DARPins.

Because the structural data indicated that the C-cap of the high-affinity DARPins is mobile in the crystals (Fig. 5a,b), we performed an ensemble of experiments to characterize the C-cap behavior of the DARPins in solution. Gel filtration analysis showed that the elution volume of A-C2 and of TM-3 is lower than that of D1 (Fig. 6a), indicating a higher hydrodynamic radius. This result is expected for proteins of similar molecular weights but with different folding states^20^. An alternative explanation, that of a different oligomeric state of the evolved DARPins compared to that of D1, was ruled out by size exclusion chromatography coupled to multi-angle laser light scattering (SEC-MALLS) analysis: both D1 and TM-3 remained monomeric under the conditions of the gel filtration experiment (Supplementary Fig. 1). We also submitted the DARPins to limited proteolysis. In the presence of subtilisin at a 1:2000 protease:DARPin molar ratio, in the case of A-C2 and of TM-3, a polyacrylamide gel electrophoresis analysis indicated that the intensity of the band corresponding to full length DARPin decreased rapidly whereas that of a band of slightly lower molecular weight increased sharply (Fig. 6b). Under the same conditions, uncleaved DARPin remained the main species in case of D1. Mass spectrometry analysis of a subtilisin-treated TM-3 sample identified a mixture of two species of about 15.8 kDa with a mass difference of 71 Da (Supplementary Fig. 2). Only a TM-3 fragment of limits 1-149 was compatible with the higher mass whereas the lower mass matched with that of fragments of limits 1-148 (one Ala residue shorter than 1-149) and 11-160. The latter solution involving two proteolytic cuts implied that, combined with the cut after residue 149, two additional fragments (1-160 and 11-149) should exist (Supplementary Fig. 2). Because these fragments were not detected by mass spectrometry, we conclude that subtilisin cleaved the TM-3 DARPin preferentially after residues 148 or 149. These cleavage sites, at the C-terminal end of the loop connecting the last internal ankyrin repeat to the C-cap helical motif (Fig. 5c), were less accessible in D1. These results point to evolved DARPins with a flexible C-cap, in agreement with the structural data. Circular dichroism analysis further indicated a lower helical content of these DARPins compared to that of D1 (Fig. 6c), therefore suggesting that their C-cap is not only mobile but also disordered.

In order to validate the result of the proteolysis experiment, we made DARPin constructs terminating after residue 149 both within the D1 framework and that of the evolved A-C2 and TM-3 DARPins. Interestingly, the circular dichroism signal of the TM-3 1-149 construct was similar to that of TM-3 and of A-C2 (Fig. 6c), indicating a similar helical content and further supporting the hypothesis that the C-cap is disordered in the evolved full length DARPins. Remarkably, an ELISA analysis showed that the removal of the C-cap of D1 led to a high-affinity DARPin, similar to A-C2 or TM-3 and their 1-149 variants (Fig. 6d), again suggesting that the C-cap interaction is detrimental to tubulin−D1 assembly. Alternatively, because the 1-149 constructs were only marginally stable and prone to aggregation, the higher signal in the ELISA experiment might result from an avidity effect of multimeric (aggregated) 1-149 constructs. However, because of the similar signal obtained with the 1-149 constructs and with evolved full length DARPins, and because in the latter case the DARPins are monomeric (Supplementary Fig. 1), this mechanism seems less likely. Moreover, it has been shown previously that C-cap-truncated DARPins remain monomeric at the concentrations used in these experiments^21^, even though the C-cap is a main safeguard against aggregation. We also studied the interaction with tubulin of DARPins devoid of a C-cap by fluorescence spectroscopy and by SPR. For the fluorescence experiments, because of the difficulties to handle the 1-149 constructs, we generated a fluorescent short construct in one step, by submitting the acrylodan-labeled TM-3 variant to limited proteolysis. The results of these experiments agree with those of ELISA and show that the affinity for tubulin of DARPins either without a C-cap (TM3 1-149 or subtilisin-digested TM-3) or with a disordered one (A-C2 and TM-3) are of the same order of magnitude (Fig. 2a, Table 1). Another important conclusion from these experiments is that the C-cap, albeit disordered, still maintains its function in preventing aggregation, since A-C2 and TM-3 are monomers in solution, as is the progenitor D1. Not unexpectedly however, the destabilization of the C-cap led to slightly less stable proteins, as evaluated by thermal denaturation (Supplementary Fig. 3).

### Destabilizing the C-cap is the major route towards higher affinity binders

**Figure 7:**
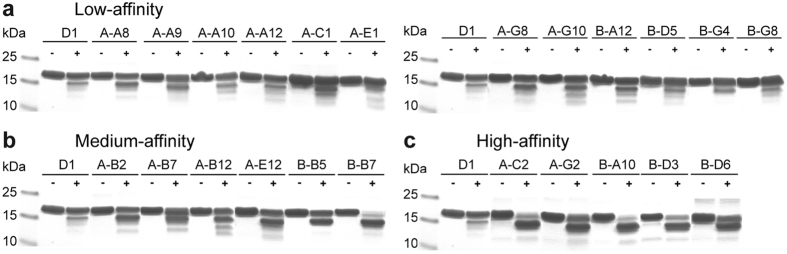
The C-cap stability of D1 mutants conditions their affinity for tubulin. SDS-PAGE analysis of limited proteolysis of DARPins in the conditions described in Fig. 6b, before (−) and after (+) incubation with subtilisin for 30 minutes. DARPins are ranked as low- (**a**) medium- (**b**) or high-affinity (**c**) tubulin binders according to Fig. 1.

We then asked whether the stability of the C-cap also correlates with the affinity for tubulin of the other variants generated from D1. As a means to characterize the C-cap stability, we recorded the resistance to proteolysis of all the variants studied in Fig. 1. The DARPins ranked as low-affinity binders were mostly resistant to limited proteolysis (Fig. 7a). The band corresponding to full-length protein remained the strongest one in all cases. In the case of the medium-affinity DARPins, the general trend was a mixture of full-length protein and cleaved fragments with bands of similar intensity on SDS-PAGE (Fig. 7b), with the exception of one DARPin (B-B7) which was less resistant to subtilisin. Finally, all the high-affinity binders were susceptible to subtilisin cleavage and behaved mainly as A-C2 (Fig. 7c). We also sequenced all these DARPins. The low-affinity ones, which were also the most resistant to proteolysis, had residue substitutions that were spread all along the sequence (Supplementary Fig. 4a). In the case of the medium-affinity DARPins, several mutations were clustered in the third internal repeat and in the C-cap (framed in Supplementary Fig. 4b), in the region expected to influence the C-cap stability. In the case of the high-affinity binders, two of them had the H118R mutation and I152T was found three times (Supplementary Fig. 4c). None of these two substitutions was found in the low- and medium-affinity DARPins. Collectively these results suggest that the affinity for tubulin is inversely correlated with the stability of the C-cap. Because the high-affinity DARPin A-G2 does not contain any of the mutations introduced in the TM-3 optimized DARPin, these results also imply that there is more than one way to destabilize the C-cap to yield high-affinity binders.

## Discussion

In this study, we identified DARPins with an improved affinity for tubulin associated with the destabilization of the C-cap. The progenitor of evolution, DARPin D1, shows electrostatic repulsions (Fig. 5c) and it would clash with the target tubulin within the C-cap, as can be deduced from the structure of the uncomplexed molecule when docked onto tubulin. The actual complex of D1 with tubulin avoids this clash by a rotation of the C-cap (Fig. 4). Evolution has brought about mutations which *destabilize* the interaction of the C-cap with the rest of the DARPin, leading to an overall affinity increase with the target. Thus, the energetic cost of removing the C-cap from its normal position has been lowered, translating into an overall better affinity with a K_D_ estimated by fluorescence measurements in the subnanomolar range (Table 1).

In uncomplexed D1, like in all DARPins, the C-cap mediates numerous interactions with the last internal repeat. Upon tubulin binding, the D1 conformational change is accompanied by the loss of many of these interactions, including the hydrogen bonds between Asn158 and the carbonyl main chain of two residues of the neighboring repeat (illustrated in Fig. 5a). Consistently, in the structure of tubulin−D1, the C-cap atomic temperature factors are substantially higher than those of the rest of the molecule (75 Å^2^ for the two C-terminal helices versus 47 Å^2^ for the N-cap motif and the three internal repeats up to residue 149)^9^. Therefore, the C-cap goes from a well-defined state in D1 to an intermediate, more mobile, state in the tubulin−D1 complex and to a disordered (Fig. 6) state in the evolved DARPins. In this last case, the negative charges of the C-cap are remote from a tubulin negative patch, unlike what is seen in tubulin-D1, providing a rationale for the gain in affinity. It remains however possible that, in the evolved DARPins, the C-cap, while too mobile to be seen in the crystal structure, contributes to the interaction by mediating dynamic contacts with tubulin. But, because the DARPins devoid of a C-cap behave as high-affinity tubulin-binders (Figs 2a and 6d, Table 1), the contribution of these putative dynamic interactions is likely to be low. Nonetheless, the presence of the disordered C-cap protects the protein against aggregation.

The three mutations introduced to generate the optimized TM-3 variant from the parent DARPin (Fig. 3a) all contribute to destabilize the C-cap. They do so either by introducing steric conflicts between the last internal repeat and the C-cap (as is the case for the H118R substitution, Fig. 5a) or by removing potential stabilizing interactions. Indeed, those mediated by Asn158 (see above and Fig. 5a) are lost due to the substitution for a Ser residue and its shorter side chain. And Ile152 points into a cavity boxed in, in particular, by Trp112, Phe145, Ile122 and by the side chain methylenes of Lys147. The more polar side chain of a Thr residue at position 152, compared to that of an isoleucine, is likely to be less favorable in this hydrophobic pocket. Interestingly, the lower stability of the original DARPin C-cap compared to internal repeats was previously noticed, and it has been shown that an Ile-to-Leu substitution at a position equivalent to position 152 of D1 reinforces the stability of these proteins in subsequent C-cap designs^21^. Conversely, our findings provide a case where an unstable C-cap is a key advantage for the identification of stronger binders, as the original version led to a repulsion with the target.

Affinity maturation is a common approach to identify high(er) affinity binders. Potentially every residue can be diversified, including those of the so-called protein framework, i.e. outside of the residues randomized in the original library and chosen to point towards typical targets. As an example, in the case of DARPins selected to bind the human epidermal growth factor receptor 2 (Her2), the mutation of 4 residues of the framework accounted for most of the gain in affinity^3^. Of particular interest, the main beneficial change was due to the substitution H52Y. This residue of the first internal repeat is at the interface with the second internal one. Structural analysis indicated that this substitution led to an opening of the anti-Her2 DARPin with a rotation of the 2^nd^ internal repeat with respect to the first one. More recently, it was shown that this open conformation is retained when this DARPin is in complex with its target and fits it very well^22^. A similar structural change is also found in D1. In this case it is induced upon tubulin binding and involves the interface between the last internal repeat and the C-cap (Fig. 4a). The residue 52 of the anti-Her2 DARPin corresponds to residue 118 of the 3^rd^ internal repeat of D1. Therefore, a mutated residue at this position in a repeat has been selected twice in affinity maturation experiments for two unrelated targets. It either induces a ready-to-interact conformation (anti-Her2 DARPin) or destabilizes the C-cap (anti-tubulin DARPin, Fig. 5a). These two pathways lead to different structural results but in both cases to improved affinities.

Protein−protein association is often accompanied by local structural changes and by domain movements, either induced fits or disorder-to-order transitions^16^. In principle, during a maturation process, evolution may improve the interaction by taking advantage of all these mechanisms. It may replace residues at the protein−protein interface, either to provide the evolved binder with chemical groups ideally positioned to interact with the partner (e.g., ref. 23) or to remove the entropic cost of stabilizing long and flexible side chains. Indeed the charged amino acids Lys, Glu and Asp are found depleted at those interfaces^24^, an observation that has been harnessed to enhance the crystallization ability of proteins through the mutation of lysine and glutamate patches^25^. Evolution may also lead to the stabilization of a ready-to-interact conformation (e.g., ref. 22) or to the optimization of loop length, which is best described in antibody maturation^26^,^27^. Order-to-disorder transitions have also been reported; in these cases the mechanism relies on the exposure of a previously hidden well-defined interacting region^7^. To the best of our knowledge, the specific mechanism found here is unprecedented. It is an order-to-disorder transition, going from an (ordered) parent binder (with a repulsion to the target) to a mature one (where one motif is disordered and the repulsion is avoided), with two orders of magnitude higher affinity, resulting in a sub-nanomolar dissociation constant. It has been brought out in an *in vitro* selection experiment. Whether this mechanism also operates in nature remains to be determined.

## Methods

### Affinity maturation, ribosome display selection and screening of high affinity DARPins

#### Affinity maturation

Affinity maturation of DARPin D1 was performed over two generations using the ribosome display selection procedure as described earlier^28^. Each generation was comprised of three rounds: (i) diversification by error-prone PCR followed by selection of all binders using ribosome display, (ii) amplification followed by incubation with competitor for slow off-rate selection under highly stringent conditions, and (iii) a rescue round to amplify and select the rare high-affinity binders.

#### Subcloning and error-prone PCR

For affinity maturation, DARPin D1 gene cloned in pDST67^9^ was subcloned in pRDV^8^,^29^, which was used as template. Error-prone PCR was performed in the presence of dNTP analogues, dPTP (6-(deoxy-β-d-erythro-pentofuranosyl)-3,4-dihydro-8H-pyrimido-[4,-5c][1,2]oxazine-7-one-5′-triphosphate) and 8-oxo-dGTP (8-oxo-2′-deoxyguanosine-5′-triphosphate)^28^. Two PCR reactions were performed using 3 and 9 μM dNTP analogues to obtain PCR products at low and high mutational loads, respectively. PCR products of both reactions were mixed in equimolar amounts and used as template for *in vitro* transcription.

#### Ribosome display selection

The random mutant library obtained by error-prone PCR was subjected to *in vitro* transcription and translation^28^. All selection steps were performed at 4 °C, except the blocking step which was performed at room temperature. For selection, 96-well MaxiSorp plates (Nunc) were coated overnight with 66 nM neutravidin or streptavidin in 50 mM Tris-HCl pH 8, 150 mM NaCl (TBS). Coated plates were washed 3 times with TBS and blocked for 1 hour with 0.5% BSA in TBS supplemented with 0.05% Tween-20 (TBST). Blocked plates were washed 3 times with TBST then biotinylated stathmin-derived peptide-coupled tubulin^9^,^13^ was incubated in 20 mM PIPES-K pH 6.8, 150 mM KCl, 50 mM Mg-Acetate, 0.05% Tween-20, and 0.1 mM GTP (buffer WBT) with 0.5% BSA for 30 minutes. For rounds 1 and 3 (diversification and rescue rounds, respectively) of both generations, 10 nM of target was used for immobilization, while for off-rate selection rounds its concentration was reduced to 1 and 0.1 nM in generations 1 and 2, respectively. After *in vitro* translation, the random mutant library of DARPin D1 was incubated with the target for 1 hour and washed five times with WBT comprising 0.1% BSA (WBT-B). From the second round onward, the translation mix containing the ternary mRNA–ribosome–DARPin complexes were first prepanned for 15 min in a well without the biotinylated target. Further, in the off-rate selection rounds the target-bound translated library was incubated with 1 μM tubulin in WBT-B for 2 hours. After incubation with the tubulin solution, the bound complex was washed thoroughly 8 times with WBT-B. RNA from selected binders was eluted with 50 mM Tris-acetate pH 7.6, 100 mM NaCl, and 25 mM EDTA and reverse-transcribed to cDNA^28^. The cDNA obtained from the first generation was amplified by 35 PCR cycles to obtain rare high affinity binders, which were subjected to diversification by error-prone PCR in the second generation. All the steps in the second generation were identical to those of the first one except that the concentration of modified tubulin submitted to immobilization for the off-rate selection round was decreased 10-fold as indicated above.

#### Screening by crude extract ELISA

For affinity analysis of individual mutants, the selected pool was cloned in plasmid pDST67 via *BamHI* and *HindIII*. All ELISA steps were performed at 4 °C except for blocking, which was performed at room temperature. MaxiSorp plates (Nunc) were coated with neutravidin, washed, blocked with BSA and were further coated with 10 nM biotinylated peptide-coupled tubulin, as described above. After washing 3 times with WBT, cell lysate of individual mutants, prepared as described earlier^28^ and diluted 1:1000 times in WBT-B, were added to the target-containing wells or to a control without immobilized tubulin. After a 1 h incubation, wells were washed with WBT and a 1:5000 dilution of an anti-RGS(H)_4_ antibody (Qiagen) in WBT-B was added and incubated for 1 h. After a washing step, a 1:20,000 dilution of a goat-Fab specific anti-mouse IgG-AP-conjugate antibody (Sigma) in WBT-B was added and incubated for 1 h. Binding was detected by monitoring the absorbance at 405 nm upon hydrolysis of disodium 4-nitrophenyl phosphate (Fluka) catalyzed by alkaline phosphatase in 50 mM NaHCO_3_, 50 mM MgCl_2_ and using an Infinite M1000 Pro microplate reader (Tecan). The amino acid sequences of the selected DARPins were obtained by DNA sequencing.

### Mutagenesis, protein purification and labeling

All site directed mutagenesis and recombination of multiple mutations were performed using a PCR-based overlap extension method^30^. Multiple mutations were introduced either by incorporating them in the same primer or using a template having the desired mutation. The introduced mutations were confirmed by DNA sequencing.

Tubulin was purified from sheep brain by two cycles of assembly and disassembly in a high molarity PIPES buffer^31^ and stored in liquid N_2_ in 50 mM Mes-K pH 6.8, 33% glycerol, 0.5 mM EGTA, 0.25 mM MgCl_2_ and 0.1 mM GTP. Prior to use, an additional cycle of microtubule assembly and disassembly was performed to remove any non-functional protein. Microtubule disassembly was performed in 15 mM PIPES-K pH 6.8, 0.4 mM MgCl_2_, 0.2 mM EGTA and 10 μM GDP. Tubulin concentration was determined spectrophotometrically using an extinction coefficient at 278 nm of 1.2 mg^−1 ^ml^−1 ^cm^−1^ assuming the molecular mass of the heterodimer is 100 kDa^32^.

DARPins were expressed in XL1-Blue cells (Agilent Technologies) grown, induced, harvested and lysed essentially as described earlier^9^. For ELISA experiments, mutant DARPins were purified in small batches of 50 ml culture volume. The resulting cell lysate was loaded on a 0.5 ml Ni-NTA agarose (Qiagen) column and washed twice with 50 mM Tris-HCl pH 8, 15 mM imidazole (TIB) and 300 mM NaCl; then a high salt wash (TIB having 1 M NaCl) was performed followed by a low salt wash (TIB having 10 mM KCl). Bound protein was eluted with 250 mM imidazole in 50 mM Tris-HCl pH 8, 150 mM KCl. Buffer was exchanged using PD-10 colums (GE Heathcare) equilibrated with 20 mM PIPES-K pH 6.8, 150 mM KCl, 1 mM MgCl_2 _and 0.5 mM EGTA (PKM buffer). For other experiments, DARPins were purified in large batches (1 L culture) using Histrap columns (GE Healthcare) followed by size exclusion chromatography on Superdex 75 (GE Healthcare) equilibrated with PKM buffer. The concentration of DARPins was estimated by UV absorption at 280 nm, using extinction coefficients calculated from the sequence with the ProtParam webserver (http://web.expasy.org/protparam). For DARPin 1-149 constructs, care was taken to remove any aggregates. In particular, just before use, purified proteins were ultracentrifuged and subjected to an additional gel filtration step. The peak corresponding to 1-149 monomers was collected and the protein concentration was determined using a bicinchoninic acid assay (BCA; Thermo Fisher Scientific) taking DARPin D1 as a reference.

DARPins were labeled with acrylodan mainly as described previously^33^. Briefly, the cysteine of Q26C mutants of D1, A-C2 and TM-3 was reduced and then reacted with acrylodan, following which the excess dye was removed by size-exclusion chromatography on a Superdex 200 10/300 GL (GE Healthcare). As for the 1-149 constructs, BCA was used to determine the concentration of labeled proteins.

### ELISA experiments

To rank the DARPin variants according to their affinity for tubulin, ELISA experiments were performed essentially as described above for the analysis of the crude extracts, with minor modifications. For lower screening stringency, 10 nM of biotinylated peptide-coupled tubulin was used for immobilization on neutravidin-coated plates, while to achieve higher stringency the concentration of modified tubulin was reduced to 2 nM. After a washing step, purified DARPins diluted to 30 nM in WBT-B were incubated with the immobilized target for binding. After washings, the target-bound DARPins were incubated for 1 h either with buffer (WBT-B) or with 100 nM tubulin to prevent the rebinding of detached DARPins. Detection was performed as described above.

### Equilibrium and kinetic analysis of tubulin–DARPin interaction by fluorescence

The dissociation constant (K_D_) at equilibrium was determined using a fixed concentration of acrylodan-labeled DARPin (either 100 nM (D1) or 15 nM (high affinity DARPins)) titrated against increasing concentration of tubulin in 20 mM K-phosphate buffer pH 7.2, 100 mM KCl, 1 mM MgCl_2_, 0.5 mM EGTA and 10 μM GDP at 20 °C. The increase in fluorescence (λ_ex_, 290 nm; λ_em_, 510 nm) was monitored in a FluoroMax spectrofluorometer (Jobin Yvon, HORIBA). The data were fitted to a 1:1 binding isotherm using Equation 1,


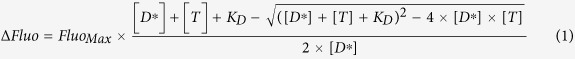


where Δ*Fluo* is the variation of the fluorescence signal, *Fluo*_*Max*_ is the fluorescence at saturating concentration of tubulin, [*T*] and [D*] are the concentrations of tubulin and labeled DARPin, respectively, and *K*_*D*_ is the dissociation equilibrium constant.

The dissociation rate constant k_off_ of D1 was determined by adding 2.75 or 5.5 μM unlabeled D1 to a 100 nM labeled D1 and 500 nM tubulin solution (final concentrations) using a SX20 Stopped-Flow spectrometer (Applied Photophysics). The same results were obtained with both concentrations of unlabeled D1 used as a chase. For the high affinity DARPins, 2 μM unlabeled DARPin was added to the fluorescent complex made of 20 nM labeled DARPin and 40 nM tubulin, and the fluorescence decrease was monitored in the spectrofluorometer. The drop in fluorescence signal was fitted with the following mono-exponential decay function,


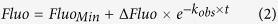


where *Fluo* is the fluorescence signal, *Fluo*_*Min*_ is fluorescence at infinite time, Δ*Fluo* is the amplitude of the fluorescence variation, and *k*_*obs*_ corresponds to k_off_ at saturating concentration of DARPin.

The association rate constant k_on_ was determined using a Hi-Tech KinetAsyst stopped-flow system (TgK Scientific). At least a 5-fold excess of tubulin was added to a fixed concentration (50 nM) of labeled DARPin, which made the binding reaction pseudo-first-order. The k_obs_ value was fitted with the following exponential equation,


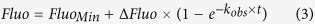


where *Fluo*_*Min*_ is the fluorescence at time 0. k_on_ was extracted from the variation of the apparent rate constant k_obs_ as a function of the tubulin concentration.

### Kinetic analysis of tubulin–DARPin interaction by SPR

Surface plasmon resonance was measured using a Proteon XPR36 instrument (Bio-Rad). The measurements were performed at 20  °C in the same buffer as the one used in the fluorescence experiments, but with a lower (20 μM) EGTA concentration and adding 0.005% Tween 20. HTG sensor chips (Bio-Rad) were used to immobilize the DARPins through their His-tag. For the determination of kinetic data, tubulin was injected at five different concentrations centered around the expected K_D_ value. The kinetic data were fitted according to the Langmuir isotherm using the Proteon Manager software. In the case of D1, although the fit was not as good as that of the data of the evolved DARPins, the different conditions tested (different amounts of immobilized D1, various tubulin concentrations, different durations of the association phase) led to very similar kinetic values. In addition, the estimation of the K_D_ obtained by plotting the RU value at the plateau as a function of tubulin concentration (Supplementary Fig. 5) agreed with the k_off_/k_on_ ratio value.

### Crystallization and structure determination

Uncomplexed D1 (not bound to tubulin) was crystallized by vapor diffusion in a buffer consisting of 100 mM Na-acetate pH 4.6, 20 mM CaCl_2_ and 30% 2-methyl-2,4-pentanediol. Crystals were flash-cooled in liquid nitrogen and diffraction data were collected at 100 K at the SOLEIL Proxima 1 beamline and processed with XDS^34^ and XSCALE^35^. The structure was solved by molecular replacement with Phaser^36^ taking the structure of a 3-variable ankyrin repeat DARPin (PDB code 2XEE^37^) as a search model. The structure was refined in Phenix^38^, alternating refinement with manual model building in Coot^39^.

Uncomplexed TM-3 was crystallized in 100 mM Na-acetate pH 4.6 and 2 M Na-formate. The crystallization buffer was supplemented with 25% glycerol as a cryo protectant and data were collected at 100 K at the ESRF ID30A-3 beamline. Data were processed with XDS^34^ and Aimless^40^ and the structure was solved with Phaser taking a C-cap truncated model of D1. The Buster program was used for refinement^41^.

To crystallize the tubulin–A-C2 complex, the C-terminal tails of tubulin were first cleaved with subtilisin^42^. A-C2 was added to digested tubulin in 1.5:1 A-C2:tubulin molar ratio. The complex was purified on a Superdex 200 10/300 GL column (GE Healthcare), equilibrated with 20 mM Hepes pH 7.2, 100 mM KCl, 1 mM MgCl_2_ and 0.5 mM EGTA. The GDP concentration was adjusted to 0.2 mM, then the complex was concentrated to 200 μM and stored in liquid N_2_ until use. Crystals grew spontaneously within 24 h in 25% (w/v) polyethylene glycol 4000 (PEG 4000), 0.1 M tri-sodium citrate pH 5.6 and 0.2 M ammonium sulfate. These crystals were used for microseeding of drops having a lower (16%) PEG 4000 concentration. Large, separate crystals were obtained within 7 days. They were frozen in the crystallization buffer supplemented with 20% glycerol as cryo protectant. Two diffraction data sets were collected on the same crystal at 100 K at the SOLEIL Proxima 1 beamline and processed, scaled and merged with XDS and XSCALE. The structure was solved by molecular replacement with Phaser using tubulin–D1 (PDB code 4DRX^9^) as a search model. The structure refinement was performed using Buster. Data collection and refinement statistics are reported in Supplementary Table 1. Coordinates and structure factors have been deposited with the Protein Data Bank with accession codes 4DUI (D1), 5EYL (TM-3) and 5EYP (tubulin–A-C2). Figures of structural models were generated with PyMOL^43^. The electrostatic potential at the tubulin–D1 interface was calculated using APBS^44^ and rendered in PyMOL.

### Characterization of the DARPins in solution

#### Analytical Gel Filtration

Samples of 100 μl purified DARPins at 100 μM concentration were loaded on Superdex 75 10/300 GL column (GE Healthcare) equilibrated with 50 mM K-phosphate pH 7.2, 150 mM KCl, 1 mM MgCl_2_ and 0.5 mM EGTA. Proteins were resolved at 0.4 ml/min flow rate using an Äkta Purifier system (GE Healthcare).

#### Limited proteolysis

Proteolysis of DARPins was performed in 20 mM Hepes pH 7.2, 100 mM KCl, 1 mM MgCl_2_ and 0.5 mM EGTA. DARPins at 40 μM were mixed with 20 nM subtilisin (Protease Type VIII; Sigma) and incubated at 25 °C. Aliquots were removed at 0, 15 and 30 minutes and the reaction was stopped by adding 0.1% (w/v) phenylmethylsulfonyl fluoride and subjected to SDS-PAGE analysis.

#### Mass Spectrometry Analysis

TM-3 DARPin was subjected to limited proteolysis for 30 minutes as described above. Post cleavage buffer was exchanged to 50 mM ammonium acetate and the sample was analyzed by electrospray ionisation mass spectrometry (ESI-MS).

#### Circular dichroism

Far-UV circular dichroism spectra of 20 μM purified DARPins (5 μM in the case of TM-3 1-149) in 50 mM Na-phosphate pH 7.2, 150 mM NaCl were recorded from 200 to 260 nm in a 1 mm path length cuvette using a J-810 Spectropolarimeter equipped with a Peltier-controlled cuvette holder (Jasco). Data were acquired with 3 accumulations at a scan speed of 100 nm/min with 0.2 nm pitch and 2 s integration time. The bandwidth was 2 nm. All spectra were buffer-subtracted and normalized and are represented as molar ellipticity. Thermal unfolding of DARPins was performed by heating 10 μM of protein from 20 to 90 °C at a rate of 0.5 °C per minute while recording ellipticity at 222 nm. Reversibility of thermal unfolding was estimated by cooling the protein samples to 20 °C followed by recording the spectra after 20 minutes of incubation. The extent of reversibility for D1 and A-C2 was 95 and 40%, respectively.

## Additional Information

**Accession codes:** Coordinates and structure factors have been deposited with the Protein Data Bank (accession code: 4DUI (D1), 5EYL (TM-3) and 5EYP (tubulin–A-C2)).

**How to cite this article**: Ahmad, S. *et al*. Destabilizing an interacting motif strengthens the association of a designed ankyrin repeat protein with tubulin. *Sci. Rep.* **6**, 28922; doi: 10.1038/srep28922 (2016).

## Supplementary Material

Supplementary Information

## Figures and Tables

**Table 1:** Equilibrium and kinetic constants of tubulin complexes with DARPin D1 and its variants.

DARPins	D1	A-C2	TM-3	C-cap-truncated TM-3
Fluorescence data				(Digested TM-3)
K_D_ (titration) (nM)	127 ± 12	0.82 ± 0.18	0.90 ± 0.19	1.03 ± 0.28
k_off_ (s^−1^)	1.49 ± 0.07	0.0071 ± 0.0002	0.0049 ± 0.0002	0.0046 ± 0.0001
k_on_ (M^−1^s^−1^)	1.30 × 10^7^	1.60 × 10^7^	1.82 × 10^7^	2.67 × 10^7^
k_off_/k_on_ (nM)	115 ± 6	0.444 ± 0.013	0.269 ± 0.013	0.172 ± 0.004
SPR data				(TM-3 1–149)
K_D_ (titration) (nM)	300 ± 30	n.d.	n.d.	n.d.
k_off_ (s^−1^)	0.22 ± 0.01	0.0041 ± 0.0001	0.0025 ± 0.0002	0.0027 ± 0.0001
k_on_ (M^−1^s^−1^)	8 ± 1.5 × 10^5^	1.2 ± 0.2 × 10^6^	1.9 ± 0.2 × 10^6^	1.7 ± 0.1 × 10^6^
k_off_/k_on_ (nM)	275 ± 75	3.4 ± 0.6	1.3 ± 0.2	1.6 ± 0.2

n.d.: not determined.
