# Supplementary material for: Destabilizing an interacting motif strengthens the association of a designed ankyrin repeat protein with tubulin
Source: Sci Rep. 2016 Jul 6;6:28922. doi: 10.1038/srep28922 (PMC4933879; doi:10.1038/srep28922)
Supplement: Supplementary Information [file srep28922-s1.pdf]

Destabilizing an interacting motif strengthens the association of a designed ankyrin repeat protein with tubulin

Shoeb Ahmad[1], Ludovic Pecqueur[1,4], Birgit Dreier[2], Djemel Hamdane[3], Magali Aumont-Nicaise[1], Andreas Plückthun[2,*], Marcel Knossow[1,*] and Benoît Gigant[1,*]

[1]Institute for Integrative Biology of the Cell (I2BC), CEA, CNRS, Univ Paris-Sud, Université Paris-Saclay, 91198, Gif-sur-Yvette cedex, France

[2]Department of Biochemistry, University of Zurich, Winterthurerstrasse 190, CH-8057, Zurich, Switzerland

[3]Laboratoire de Chimie des Processus Biologiques, CNRS-UMR 8229, Collège De France, 11 place Marcelin Berthelot, 75231 Paris Cedex 05, France

[4] Present address: Laboratoire de Chimie des Processus Biologiques, CNRS-UMR 8229, Collège De France, 11 place Marcelin Berthelot, 75231 Paris Cedex 05, France

*Correspondence should be addressed to A.P. (plueckthun@bioc.uzh.ch) or M.K. (marcel.knossow@i2bc.paris-saclay.fr) or B.G. (benoit.gigant@i2bc.paris-saclay.fr)

Supporting Information

Supplementary Table 1

Supplementary Figures 1 to 6

Supplementary Table 1. Data collection and refinement statistics for D1, TM-3 and tubulin−A-C2 complex structures.

	D1	TM-3	Tubulin−A-C2
**Data collection***			
Space group	$P4_12_12$	H32	$P2_1$
Cell dimensions			
a, b, *c* (Å)	43.4, 43.4, 143.1	105.3, 105.3, 193.0	87.6, 71.8, 93.0
α, β, γ,°	90.0, 90.0, 90.0	90.0, 90.0, 120.0	90.0, 99.85, 90.0
Resolution (Å)	41.53 - 1.16 (1.23 - 1.16)	52.7 - 2.41 (2.50 - 2.41)	49.74 - 1.90 (1.95 - 1.90)
R_{meas}	0.063 (0.377)	0.145 (1.36)	0.106 (2.002)
$I / \sigma I$	14.17 (2.96)	10.4 (1.6)	11.85 (1.01)
$CC_{1/2}$	99.8 (87.5)	99.8 (55.5)	99.8 (36.0)
Completeness (%)	99.5 (97.7)	99.4 (96.0)	100.0 (100.0)
Multiplicity	5.1 (3.8)	6.7 (6.4)	9.0 (9.0)
**Refinement**			
Resolution (Å)	32.1 - 1.16	44.4 - 2.41	49.74 - 1.90
No. reflections	48392	16079	89661
R_{work} / R_{free}	0.123 / 0.149	0.174 / 0.219	0.173/0.208
No. atoms			
Protein	1380	2018	7693
Ligand/ion	32	29	153
Water	167	85	655
B factors			
Protein	11.5	57.8	48.4
Ligand/ion	18.8	90.6	53.4
Waters	24.9	62.0	55.6
Coordinate error (Å)	0.12	0.306	0.280
R.m.s.d.			
Bond lengths (Å)	0.013	0.010	0.010
Bond angles (°)	1.24	1.21	1.05
Ramachandran			
Favored region (%)	98.28	100	97.05
Allowed region (%)	1.72	0	2.75
Outliers (%)	0	0	0.20

*Data were collected on a single crystal. Values in parentheses are for the highest-resolution shell.

Supplementary Figures

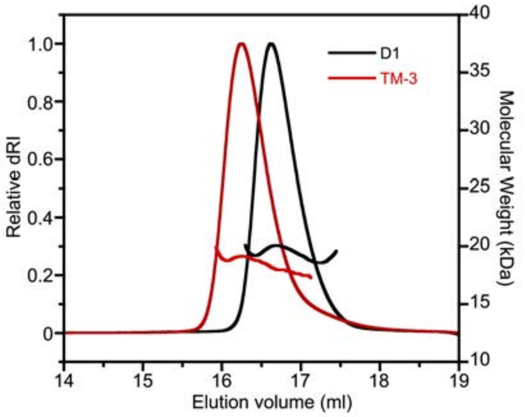

Supplementary Figure 1. **Size exclusion chromatography coupled to multi-angle laser light scattering (SEC-MALLS) analysis of D1 and of TM-3**. The differential refractive index (normalized dRI, left axis) and molecular mass (right axis) are plotted as a function of the column elution volume. The gel filtration step was carried out on a Prominence HPLC system (Shimadzu) using a Superdex 200 Increase 10/300 GL column (GE Healthcare) and with the same buffer as that of the gel filtration experiment of Fig. 6a. Samples of 100 µl DARPin at a concentration of 2 mg/ml were run at a 0.5 ml/min flow rate. Detection was performed using a three-detector static light-scattering apparatus (MiniDAWN TREOS, Wyatt Technology, equipped with a quasi-elastic light-scattering module) and a refractometer (Optilab T-rEX, Wyatt Technology). Molecular weight calculations were performed with the ASTRA 6 software (Wyatt Technology) using a dn/dc value of 0.183 ml/g. The derived masses from the static light-scattering data are 20.2 ± 0.7 kDa (D1) and 19.6 ± 0.6 kDa (TM-3), close to the calculated mass (18.0 kDa) of monomeric DARPins.

**Determined mass** (Da)	**Matching peptides**	**Theoretical mass** (Da)	**Δ mass** (Da)
15746	11-160<br>1-148	15744.808<br>15746.951	-1.192<br>0.951
15817	1-149	15818.030	1.030

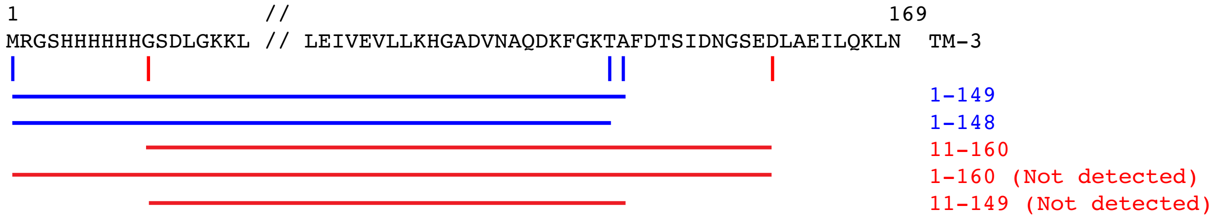

Supplementary Figure 2. Mass spectrometry analysis of subtilisin-digested TM-3 DARPin. (Top) Mass of stable fragments of subtilisin-digested TM-3 DARPin as determined by mass spectrometry and boundaries of matching fragments with their theoretical molecular weight. (Bottom) Sequence of TM-3 and putative cleavage sites (see Fig. 3a for a complete TM-3 sequence). Given that the higher determined mass corresponds to that of the 1-149 fragment, the ambiguity on the nature of the fragment of mass 15746 Da is resolved since the 1-160 and 11-149 fragments were not detected by mass spectroscopy. Therefore the TM-3 positions 11 and 160 are not preferential subtilisin-cleavage sites, hence the lower determined mass corresponds to that of the 1-148 fragment.

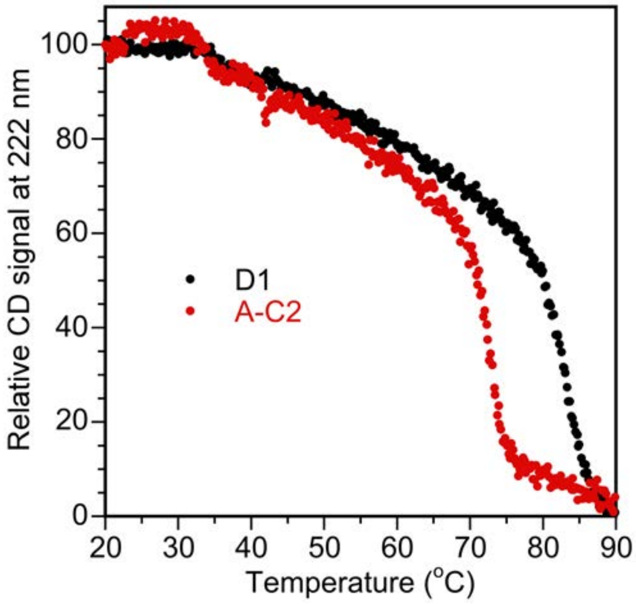

Supplementary Figure 3. Thermal unfolding profile of D1 and A-C2 DARPins recorded by circular dichroism (CD). The apparent melting temperatures of D1 and A-C2 were estimated to be 82.8 and 72.2 °C, respectively.

a

```
D1     MRGSHHHHHHGSDLGKKLLEAARAGQDDEVRILMANGADVNATDASGLTPLHLAATYGHL  60
A-A8   MRGSHHHHHHGSDLGKRLLEAARAGQDDEVRILMANGADVNATDASGLTPLHLAATYGHL  60
A-A9   MRGSHHHHHHGSDLGRKLLEAARAGQDDEVRILMANGADVNATDASGLTPLHLAATYGHL  60
A-A10  MRGSHHHHHHGSNLGKKLLEAARAGQDDEVRILMANGADVNATDASGLTPLHLAATYGHL  60
A-A12  MRGSHHHHHHGSDLGKKLLEAARAGQDDEVRILMANGADVNATDASGLTPLHLAATYGHL  60
A-C1   MRGSHHHHHHGSDLGKKLLEAARAGQDDEVRILMATGADVNATDASGLTPLHLAATYGHL  60
A-E1   MRGSHHHHHHGSDLGKKLLEAARAGQDDEVRTLMANGADVNATDASGLTPLHLAATYGHL  60
A-G8   MRGSHHHHHHGSDLGKKLLEAARAGQDDEVRILMANGADVNATDASGLTPLHLAATYGHL  60
A-G10  MRGSHHHHHHGSDLGKKLLEAARAGQDDEVRILMANGADVNATDASGLAPLHLAATYGHL  60
B-A12  MRGSHHHHHHGSDLGKKLLEAARAGQDDEVRILMANGADVNATDASGLTPLHLAATYGHL  60
B-D5   MRGSHHHHHHGSDLGKRLLEAARAGQDDEVRILMANGADVNATDASGLTPLHLAATYGHL  60
B-G4   MRGSHHHHHHGSDLGKKLLEAARAGQGDEVRILVANGADVNATDASGLTPLHLAATYGHL  60
B-G8   MRGSHHHHHHGSDLGKKLLEAARAGQDDEVRILMANGADVNATDASGLTPLHLAATYGHL  60

D1     EIVEVLLKHGADVNAIDIMGSTPLHLAALIGHLEIVEVLLKHGADVNAVDTWGDTPLHLA 120
A-A8   EIVEVLLKHGADVNAIDIMGSTPLHLAALVGHLEIVEVLLKHGADVNAVDTWGDTPLHLA 120
A-A9   EIVEVLLKHGADVNAIDIMGSTPLHLAALIGHLEIVEVLLMHGADVNAVDTWGDTPLHLA 120
A-A10  EIVEALLKHGADVNAIDIVGSTPLHLAALIGHLEIVEVLLKHGADVSAVDTWGDTPLHLA 120
A-A12  EIVEVLLKHGADVNAIDITGSTPLHLAALIGHLEIIEVLLKHGADVNAVDTWGDTPLHLA 120
A-C1   EIVGVLLKHGADVNAIDIVGSTPLHLAALIGHLGIVEVLLKHGADVNAVDTWGDTPLHLA 120
A-E1   EIVEVLLRHGADVNAIDIVGSTPLHLAALVGHLEIVEVLLKHGADVNAVDTWGDTPLHLA 120
A-G8   EIVEVLLKHGADGNAIDIMGSTPLHLAALIGHLEIVEVLLKHGADVNAVDTWGDTPLHLA 120
A-G10  EIVEALLKHGADVNAIDITGSTPLHLAALIGHPEIVEVLLKHGADVNAVDTWGDTPLHLA 120
B-A12  EIVEVLLKHGADVNAIDIMGSTPLHLTALTGRLEIVEVLLKHGADVNAVDTWGDTPLHLA 120
B-D5   EIVEVLLKHGADVNAIDIMGSTPLHLAALIGHLGIVEVLLKHGAGVSAVDTWGDTPLHLA 120
B-G4   EIVEVLLRHGADVNAIDAMGSTPLHLAALIGHLEIVEVLLKHGADVNAVDTWGDTPLHLA 120
B-G8   EIVEVLLRHGADVNAIDIMGSTPLHLAALIGHLEIVEVLLKHGADVNAVDTWGDTSLHLA 120

D1     AIMGHLEIVEVLLKHGADVNAQDKFGKTAFDISIDNGNEDLAEILQKLN 169
A-A8   AIMGHLEIVEVLLKHSADVNAQDKFGKTAFDISIDNGNEDLAEILQKLN 169
A-A9   AIMGHLEIVEVLLKHGADVNAQDKFGKTASDISIDNGDEDLAEILQKLN 169
A-A10  AIMGHLEIVEVLLRHGADVNAQDRFGKTAFDISIDNGNEDLAEILQKLN 169
A-A12  AIMGHLEIVEVLLKHGADVNAQDKFGKTAFDISIDNGNEDLAEILQKLN 169
A-C1   AIMGHLEVVEVLLKHGADANAQDKFGKTAFDISIDNGNEDLAEILQKLN 169
A-E1   AIMGHLEIVEVLLRHGADVNAQDKFGKTAFDVSIDNGNEDLAEILQKLN 169
A-G8   AIMGHLEIVEVLLKHGADVNTQDKFGKTAFDISIDNGNEDLAEILQKLN 169
A-G10  AIMGHLEIVEVLLKHGADVNAPDKFGKTAFDISIDSGNEDLAEILQKLN 169
B-A12  AIMGHLEIVEVLLKHGADVNAQDKFGKTAFDISIDNGNEDLAEILQKLN 169
B-D5   AIMGHLEIVEVLLKHGADVNAQDKFGKTAFDISIDNGNEDLAEILQKLN 169
B-G4   AIMGHLEIVEVLLKHGADVSAQDRFGKTAFDISIDNGNEDLAEILQKLN 169
B-G8   AIMGHLEIVEVLLKHGADVNAQDKFGKTALDISIDNGNEDLAEILQKLN 169
```

Supplementary Figure 4 (continued on next page)

b

```
D1    MRGSHHHHHHGSDLGKKLLEAARAGQDDEVRILMANGADVNATDASGLTPLHLAATYGHL  60
A-B2  MRGSHHHHHHGSDLGKKLLEAARAGQDDEVRILMANGADVNATDASGLTPLHLAATYGHL  60
A-B7  MRGSHHHHHHGSDLGKKLLEAARAGQDDEVRILVANGADVNATDASGLTPLHLAATYGHL  60
A-B12 MRGSHHHHHHGSDLGKKLLEAARAGQDDEVRILMANGADVDAFDSTGQTPLHLAATYGHL  60
A-E12 MRGSHHHHHHGSDLGKKLLEAARAGQDDEVRILMANGADVNATDASGLTPLHLAATYGHL  60
B-B5  MRGSHHHHHHGSDLGKKLLEAARAGQDNEVRILMANGADVNATDASGLTPLHLAATYGHL  60
B-B7  MRGSHHHHHHGSDLGKKLLEAARAGQDDEVRILMANGADVNATDASGLTPLHLAATYGHL  60

D1    EIVEVLLKHGADVNAIDIMGSTPLHLAALIGHLEIVEVLLKHGADVNAVDTWGDTPLHLA 120
A-B2  EIVEVLLKHGADVNAIDIMGSTPLHLAALIGHLEIVEVLLKHGADVNAVDTWGDTPLHLA 120
A-B7  EIVEVLLKHGADVNATDIMGSTPLHLAALIGHLEIVEVLLKHGADVNAVDTWGDTPLHLA 120
A-B12 EIVEVLLRHGADVNAIDIMGSTPLHLAALIGHLEIVEVLLKHGAGVNAVDTWGDTPLHLA 120
A-E12 EIVEVLLKHGADVNAIDTMGSTPLHLAALIGHLEIVEVLLKHGADVNAVDTWGDTPLHLA 120
B-B5  GIVEVLLEHGADVNAIDIMGSTPLHLAALIGHLKIVEVLLKHGADVNAVDTWSDTPLHLA 120
B-B7  GIVEVLLKHGADVNAIDIMGSTPLHLAALIGHLEIVEVLLKHGVDVNAVDTWGDTPLHLA 120

D1    AIMGHLEIVEVLLKHGADVNAQDKFGKTAFDISIDNGNEDLAEILQKLN 169
A-B2  AIMGHLEIVEVLLKHGAGVNAQDKLGKTAFDVAIDNGNEDLAEILQKLN 169
A-B7  AIMGHPEIVEVLPKHGADVNAQDKLGRTAFDVSIDDGNEDLAEILQKLN 169
A-B12 AIMGHLEIVEVLLKHGADVNTQDKFGKTAFDISIGNGNEDLAEILQKLN 169
A-E12 AIMGHLEIVEALLKYGADVNAQDKFGKTALDILIDNGNEDLAEILQKLN 169
B-B5  AIMGHPEIVEVLLKHGADVNTQDKFGKTAFDISIDNGNEDLAEILQKLN 169
B-B7  AIMGHPEVVEVLLRHDADVNAQDKFGKTAFDISIDNGNEDQAEILQKLN 169
```

c

```
D1    MRGSHHHHHHGSDLGKKLLEAARAGQDDEVRILMANGADVNATDASGLTPLHLAATYGHL  60
A-C2  MRGSHHHHHHGSDLGKKLLEAARAGQDDEVRVLMANGADVNATDASGLTPLHLAATYGHL  60
A-G2  MRGSHHHHHHGSDLGKKLLEAARAGQDGEVRILIANGADVNATDASGLTPLHLAATYGHL  60
B-A10 MRGSHHHHHHGSDLGKKLLEAARAGQDDEVRILMANGADVNATDASGLTPLHLAATYGHL  60
B-D3  MRGSHHHHHHGSDLGKKLLETARAGQDDEVRVLVANGADVNATDASGLTPLHLAATYGHL  60
B-D6  MRGSHHHHHHGSDLSKKLLEAARAGQDDEVRILMANGADVNATDASGLTPLHLAATYGHL  60

D1    EIVEVLLKHGADVNAIDIMGSTPLHLAALIGHLEIVEVLLKHGADVNAVDTWGDTPLHLA 120
A-C2  EIVEVLLKHGADVSASDLMGSTPLHLAALIGHLEIVEVLLKHGADVNAVDTWGDTPLRLA 120
A-G2  EVVEVLLKHGADVNAIDIMGSTPLHLAALIGHLEIVEVLLKHGADVSAVDTWGDTPLHLA 120
B-A10 EIVEVLLKHGADVNAIDTMGSTPLHLAALIGHLEIVEVLLKHGADVNAVDTWGDTPLHLA 120
B-D3  GIIEVLLKHGADVNAIDIMGSTPLHLAALTGRLEIVEVLLKHGADVNAVDTWGDTPLRLA 120
B-D6  GIVEVLLQHGADVNTIDIMGSTPLHLAALIGHLEIVEVLLKHGADINAVDTWGDTPLHLA 120

D1    AIMGHLEIVEVLLKHGADVNAQDKFGKTAFDISIDNGNEDLAEILQKLN 169
A-C2  AVMGHLKIVEALLKHGADVNAQDKFGKTAYDTSIDNGSEDLAEILQKLN 169
A-G2  AIMGHLEIVEVLLKHGADVNAQDKFGKTAFDIPTDNGNEDLAEILQKLN 169
B-A10 AIMGHLETVEVLLKHGAGVNAQDKFGKTAFDTSIDNGNEDLAEILQKLN 169
B-D3  AIMGHLEIVEVLLKHGADVSAQNQFGKTAFDISIDNGNEDLAEILQKLN 169
B-D6  AIMGHLEVVGVLLKHGADVSAQDKFGKTAFGTSIDNGNEDLAEILQKLN 169
```

Supplementary Figure 4. Multiple sequence alignments of low- (panel a), medium- (b) and high-affinity (c) mutants, colored by repeats as in Fig. 3a. In the case of the medium-affinity mutants, several mutations are clustered in the last internal repeat and in the C-cap (framed). In the case of the high-affinity mutants, the positions 118 and 152 are framed in red. The optimized TM-3 variant has mutated residues at these two positions (Fig. 3a). Two other regions gathering several mutations, in the last internal repeat and in the C-cap, are also framed (in black).

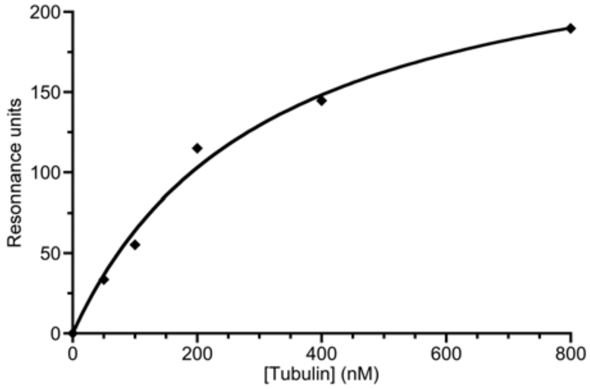

Supplementary Figure 5. **Dissociation constant of the tubulin-D1 complex estimated by SPR**. The resonance unit value at the plateau is plotted as a function of the tubulin concentration injected on a sensor chip with immobilized D1 (data from Fig. 2d, left panel). The data were fitted with the Proteon Manager software and gave a K_D value of 300 (±30) nM.

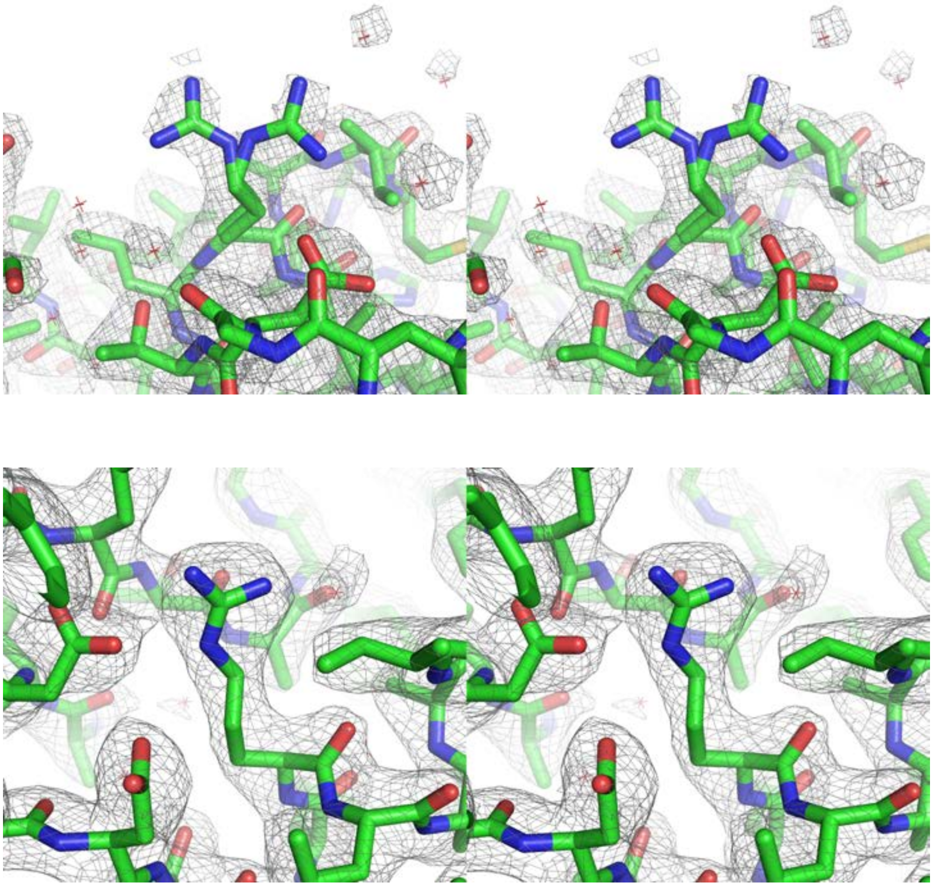

Supplementary Figure 6. Stereo views of the TM-3 2F_{obs}-F_{calc} electron density map, contoured at the 1 σ level and centered on Arg118. Top: molecule A, two alternate conformations of Arg118 have been modeled. Bottom: molecule B, in which Arg118 adopts only one conformation.
